# Editorial: Next-generation technologies and multidisciplinary integration for oral cancer diagnosis and treatment

**DOI:** 10.3389/froh.2026.1870105

**Published:** 2026-05-28

**Authors:** Abhijit Chakraborty, Abhishek Shankar, Faisal Aziz

**Affiliations:** 1 Department of Investigational Cancer Therapeutics (ICT), University of Texas MD Anderson Cancer Center, Houston, TX, United States; 2 Department of Radiation Oncology, Dr BR Ambedkar Institute Rotary Cancer Hospital, All India Institute of Medical Sciences, New Delhi, India; 3 The Hormel Institute, University of Minnesota Twin Cities, Austin, MN, United States

**Keywords:** artificial intelligence, liquid biopsy, multiomics, oral oncology, precision medicine

## Introduction

Oral cancer remains a major source of global morbidity and mortality in both high-income and resource-limited healthcare settings. Oral squamous cell carcinoma (OSCC) accounts for approximately 90% of all oral malignancies (1, 2). According to GLOBOCAN 2022, there were an estimated 389,485 new cases and 188,230 deaths due to lip and oral cavity cancer worldwide (3). Despite decades of incremental therapeutic progress, five-year survival rates have remained largely stagnant, primarily due to late-stage diagnosis, high rates of locoregional recurrence, and biological heterogeneity of the disease (1, 4).

This Research Topic was developed to examine how next-generation technologies, including artificial intelligence (AI), machine learning (ML), liquid biopsy, advanced imaging, and molecular profiling, are reshaping the landscape of oral cancer care and how multidisciplinary integration provides the framework necessary to translate these advances into clinical practice. Increasingly, progress in oral oncology is not defined by isolated innovations but by the convergence of technological advancements and coordinated clinical expertise. The four contributions in this Research Topic reflect this vision by spanning biomaterial science, surgical innovation, multidisciplinary oncologic care, and computational pathology.

Walia et al. examined the effects of clinically relevant doses of ionizing radiation on three dental restorative materials: a bulk-fill composite (3M™ Filtek™ Bulk Fill), a nanohybrid composite (Charisma Topaz One), and an alkasite-based restorative material (Cention N). Ninety disc-shaped specimens were irradiated using two fractionation protocols (70 Gy in 35 fractions and 45 Gy in 5 fractions). Vickers microhardness testing and scanning electron microscopy revealed a statistically significant decrease in surface microhardness and micromorphological degradation (*p* < 0.05) in all materials. The bulk-fill composite exhibited the greatest resistance to aging-related changes. These findings emphasize the importance of collaboration between oncologists and dental professionals in planning head and neck radiotherapy, considering the significant morbidity associated with postradiation tooth extractions.

Qi et al. refined the nasolabial flap for total lower lip reconstruction after malignant tumor excision, addressing microstomia and its functional limitations. Two elderly male patients with squamous cell carcinoma of the lower lip underwent bilateral full-thickness strip flap reconstruction using the buccal mucosa for vermilion reconstruction. At 12 months, both patients had normal mouth opening, maintained oral competence, satisfactory aesthetics, and no tumor recurrence. Patient-reported outcomes using the Visual Analog Scale indicated preserved mastication, speech, and swallowing functions. The nasolabial flap is a practical and reliable option for reconstructing full-thickness lower lip defects after oncologic resection surgery.

Valentini et al. reported a rare case of HPV/EBV-negative LEC of the right palatine tonsil in a 62-year-old woman with metachronous squamous dysplasia and an incidentally identified granular cell tumor (Abrikossoff's tumor) of the true vocal cord. The simultaneous management of these two pathologically distinct neoplasms requires input from otolaryngology, head and neck surgery, radiology, pathology, and oncology. Advanced imaging and narrow-band imaging-assisted endoscopy led to transoral robotic surgery, radical neck dissection, and adjuvant chemoradiotherapy for LEC, while the granular cell tumor was managed by close-margin excision under surveillance. This case highlights the importance of a multidisciplinary tumor board in synthesizing complex diagnostic data and orchestrating individualized therapeutic strategies for rare tumors. These cases are relevant to the increasing focus on training and disease management with robotic surgery, especially in low- and middle-income countries.

Wang et al. developed a semi-supervised deep learning framework for segmenting oral cavity-derived cancer image data. Recognizing the labor-intensive nature of fully supervised models, they proposed a method that integrates transformation-based uncertainty estimation, multi-scale contrastive learning, and a boundary-aware enhanced U-Net. Evaluated on the hematoxylin and eosin-stained OCDC dataset, the framework outperformed both fully supervised baselines and state-of-the-art semi-supervised methods on the Dice and Jaccard indices while reducing the boundary segmentation error. Notably, the model trained with only 30% of the labeled data surpassed its fully supervised counterparts trained with 100%, highlighting its potential to reduce the annotation burden and accelerate AI deployment for OSCC detection in routine pathology workflows.

Taken together, these contributions illustrate that modern oral cancer care is inherently multidimensional. No single discipline or innovation is sufficient; rather, meaningful progress arises from the alignment of surgical expertise, material science, and coordinated evidence-based clinical decision-making.

## Future directions

The future of oral oncology will be shaped by the integration of artificial intelligence, biomarker-driven precision medicine, and data-centric clinical ecosystems to improve patient outcomes. The challenge is no longer technological capability, but effective clinical integration across diverse settings. Liquid biopsy is particularly promising, as circulating tumor DNA (ctDNA), salivary microRNA signatures, exosomal RNA, and cell-free DNA from biofluids offer noninvasive early detection and real-time monitoring (5, 6). Saliva-based tests are especially appealing for oral cancer diagnosis, given their simplicity, low cost, and repeatability (7). However, broader adoption depends on standardized assays and validation across diverse populations.

AI continues to reshape diagnosis and treatment, with tools already improving disease detection and classification using imaging, digital pathology, and intraoral photographs (8, 9), as exemplified by the semi-supervised segmentation approach in this Research Topic. Beyond lesion identification, AI can also integrate multimodal datasets combining clinical, imaging, and molecular information to generate predictive models for treatment response, recurrence risk, and functional outcomes (10); it should support rather than replace clinical judgment (11). Multiomics approaches further strengthen the precision medicine paradigm by integrating genomic, transcriptomic, proteomic, and metabolomic profiling, enabling a deeper characterization of tumor heterogeneity and biological behavior (12). Digital health, telemedicine, and remote monitoring tools strengthen survivorship care (13).

Despite these advances, challenges remain. AI requires robust validation across diverse populations, regulatory clarity, and clinician acceptance. Biomarker research must shift from discovery to clinically actionable tools. Equity is crucial, as the oral cancer burden is higher in low- and middle-income countries with limited access to diagnostics and advanced care. Emerging technologies, such as AI-driven screening, label-efficient learning, smartphone diagnostics, and saliva assays, should be developed for scalability, cost-effectiveness, and accessibility. These efforts ensure broader implementation and reduce global inequities in oral cancer detection and treatment.

## Conclusion

The message of this research topic is both urgent and optimistic: oral cancer care has been constrained by conventional approaches, but next-generation technologies within a multidisciplinary framework are reshaping it. The critical barrier is the effective translation of these findings into routine clinical practice. Progress in oral oncology will result from coordinated developments across the surgical, diagnostic, computational, and translational domains. Advances in resilient biomaterials, reconstructive techniques, AI-driven diagnostics, label-efficient deep learning, and biomarker discovery are shifting toward precise, patient-centered care. The impact will be defined by enabling earlier detection, personalized treatment, improved functional outcomes, and quality of life. This is the promise and responsibility of integrated oral cancer care.
